# *H3 K27M*-mutant gliomas in adults vs. children share similar histological features and adverse prognosis 

**DOI:** 10.5414/NP301085

**Published:** 2018-02-02

**Authors:** Bette K. Kleinschmidt-DeMasters, Jean M. Mulcahy Levy

**Affiliations:** Departments of; 1 Pathology,; 2 Neurology,; 3 Neurosurgery,; 4 Pharmacology, and; 5 Pediatrics, University of Colorado School of Medicine, and; 6 The Morgan Adams Foundation Pediatric Brain Tumor Research Program, Children’s Hospital Colorado, Aurora, CO, USA

**Keywords:** pilocytic astrocytoma, ganglioglioma, midline, pons, elderly

## Abstract

Background: *H3 K27M* mutation was originally described in pediatric diffuse intrinsic pontine gliomas (DIPGs), but has been recently recognized to occur also in adult midline diffuse gliomas, as well as midline tumors with other morphologies, including gangliogliomas (GGs), anaplastic GGs, pilocytic astrocytomas (PAs), and posterior fossa ependymomas. In a few patients with *H3 K27M-*mutant tumors with these alternate morphologies, longer survival has been reported, making grading difficult for the neuropathologist. Few series compare tumors in adult vs. pediatric cohorts; we report our 4-year experience. Materials and methods: Text Word database searches using “H3 K27M” in reports generated between January 2013 and November 10, 2017 were used to identify patients. Clinical and histological features as well as survival were evaluated for each case. Results: 28 *H3 K27M*-mutant tumors were identified, with equal numbers of adults (13) vs. children (15). For adults, mean and median age was 52 years (range = 27 – 81 years), 2 decades older than a recently-published adult series. Tumors involved thalamic (adult = 7; pediatric = 7), spinal cord (adult = 4; pediatric = 2), pons (adult = 1; pediatric = 6), and hypothalamic (n = 1) sites. Other morphologies at presentation included pure GG (n = 3, pediatric) and PA (n = 1, adult). One adult and 1 pediatric patient each presented with leptomeningeal dissemination or developed leptomeningeal dissemination within 1 year after diagnosis, with transformation from PA or GG histology to glioblastoma. Mean survival was 9.3 (adults) vs. 8.9 (pediatric) months. Patients with tumors of other morphologies (GG, PA) did not enjoy extended survival. Conclusion: *H3 K27M*-mutant tumors can affect patients at advanced ages, may show leptomeningeal dissemination at time of presentation, and “pure” GG or PA morphology is not rare. Regardless of patient age or tumor morphology, patients fare equally poorly.

## Introduction 

*H3 K27M* mutations were first described in pediatric diffuse pontine gliomas (DIPGs) [1], but soon thereafter were found in midline gliomas in adults by our group [2] and Solomon et al. [3]. The presence of *H3 K27M* mutation in DIPGs was recognized to portend an adverse prognosis regardless of the histological grade of the lesion [4] and thus a grade of IV was assigned by the 2016 World Health Organization [5]. 

Although *H3 K27M* mutations were originally identified in diffuse infiltrating gliomas with astrocytic morphology in the pons, a large series of 47 cases documented that tumors can show a wide range of morphological features and can occur in spinal cord, thalamus, hypothalamus, and pineal region [3]. In that series, mutant gliomas were described with giant, epithelioid, or rhabdoid cells, as well as primitive neuroectodermal-like (PNET-like) foci, neuropil-like islands, ependymal areas, sarcomatous transformation, ganglionic differentiation, or pilomyxoid or pleomorphic xanthoastrocytoma-like areas [3]. 

More of a nomenclature/grading problem for the neuropathologist are the *H3 K27M-*mutant tumors with “pure” histologies other than diffuse infiltrating astrocytoma. In the past, these have been published almost exclusively under the diagnosis made by histological appearance and some of these patients have enjoyed several years of survival before undergoing recurrence and eventual demise. These have included a midline, histologically-classic “pure” pilocytic astrocytoma [6] and ganglioglioma [7] with *H3 K27M* mutation, but such cases at the time were considered quite exceptional [8]. Notably, in the 2013 survey study of 338 different types of pediatric CNS tumors conducted by Gielen et al. [8], 10 of 10 gangliogliomas, 15 of 15 pilocytic astrocytomas, 5 of 5 pilomyxoid astrocytomas, and 6 of 6 anaplastic pilocytic astrocytomas tested for *H3 K27M *mutation were negative. 

Since then, an increasing number of cases with pure pilocytic astrocytoma (PA) or ganglioglioma (GG) morphology have been published [9, 10, 11, 12, 13, 14], including from our own group [15]. Indeed, in a report on 18 patients with anaplastic GGs (13 adults; 5 children), *H3 K27M* mutation was found in 17% of cases, and thus anaplastic GGs appear particularly likely to show this mutation [13]. However, this mutation is not restricted to PA or GG morphologies, and other very recent case reports document *H3 K27M*-mutant gliomas with “subependymoma-like” [9] or “PNET-like” appearance and neuropil islands [16]. Rare posterior fossa ependymomas, group A, [17, 18] and adult cerebellar high-grade gliomas [19] carrying the *H3 K27M* mutation have also been published. 

Thus, although this is not a specific recommendation in WHO 2016 [5], it has become standard practice at our institution (and many others) that every midline glial tumor, regardless of patient age or histological appearance, is assessed by immunohistochemistry (IHC) for this mutation. While genetic testing for this histone gene mutation is not available in many laboratories, fortunately, a high-fidelity IHC marker has been validated that parallels the mutation [3, 20]. 

In terms of prognosis, not only are there only case reports on *H3 K27M*-mutant tumors with other morphologies, few studies have addressed outcome in adult cohorts [10, 19]. Meyronet et al. [10] found that, compared with IDH/H3 wild-type gliomas, *H3 K27M-*mutant gliomas were diagnosed at an earlier age (32 vs. 64 years, p < 0.001), always had a midline location (21/21 vs. 21/130, p < 0.001), but did not show a dramatically different median survival (19.6 months in *H3 K27M-*mutant gliomas vs. 17 months in IDH/H3 wild-type gliomas). 

We have been utilizing the H3 K27M IHC for assessment of midline glial tumors from our joint adult and pediatric neuropathology practice since 2013. We now extend our previous studies with *H3 K27M-*mutant tumors [2, 15], focusing on comparing features in these two cohorts in terms of histological appearance, leptomeningeal dissemination, and survival. 

## Materials and methods 

Text Word database searches using the term “H3 K27M” were utilized to identify cases with positive H3 K27M IHC expression in our Pathology and Pediatric Neuro oncology databases. Study dates were January 2013 to November 10, 2017. Demographic and survival information was extracted from patient medical records and histological features were available for review from the slides retained within pathology department files. The study was undertaken in compliance with institutional review board guidelines, under COMIRB #95-100. 

For histological assessment, biopsy/autopsy tissues were fixed in 10% buffered formalin and embedded in paraffin wax. Paraffin blocks were cut at 4 microns. H & E sections were mounted on charged slides (Superfrost plus side, Fisher Scientific, Pittsburgh, PA, USA) while immunohistochemical and special stains were mounted on charged slides (TOMO, Matsunami Glass Ind., Ltd., Bellingham, WA, USA). Immunohistochemistry assessed included nuclear p53 expression (DAKO Corp., Carpinteria, CA, USA), retention or loss of nuclear ATRX (α-thalassemia/mental retardation syndrome X-linked) immunostaining (using the recommended Sigma-Aldrich antibody (St. Louis, MO, USA) and Ventana benchmark processing (10)), MIB-1 (catalog number 796-4286, dilution: RTU, clone: Ki-67, mouse, Ventana Medical Systems, Tucson, AZ, USA) and H3F3A K27 (Millipore, Temecula, CA, USA). Scoring was subjective for IHC. Standard methodology fluorescence in situ hybridization was utilized for *EGFR* amplification and loss of *PTEN*/10 centromere. Given the fact that 50% of diffuse midline gliomas, *H3 K27M-*mutant, have been reported to show *TP53* mutation, which is often paralleled by increased p53 nuclear immunostaining [5], we also were interested to see if adult vs. pediatric tumors differed in this feature. Presence or absence of nuclear ATRX loss had been assessed for the original diagnosis in ~ 1/2 of these cases. Small biopsy tissue size, poor tissue fixation, and unavailability of blank slides for testing older cases limited the numbers tested. 

## Results 

Table 1 summarizes the ages, gender, anatomical location, initial histological diagnoses, and p53 IHC labeling indices discerned prior to H3 K27M IHC in the 28 *H3 K27M*-mutant tumors identified in our databases. There were with equal numbers of adults (13) vs. children (15). There were 9 females and 4 males in the adult group and 8 females and 7 males in the pediatric group. Adult ages ranged from 28 to 81 years, with mean and median age of 52 years. Pediatric ages ranged from 3 to 16 years, with a mean of 8.8 years and median of 8 years. 

**Figure 1.**
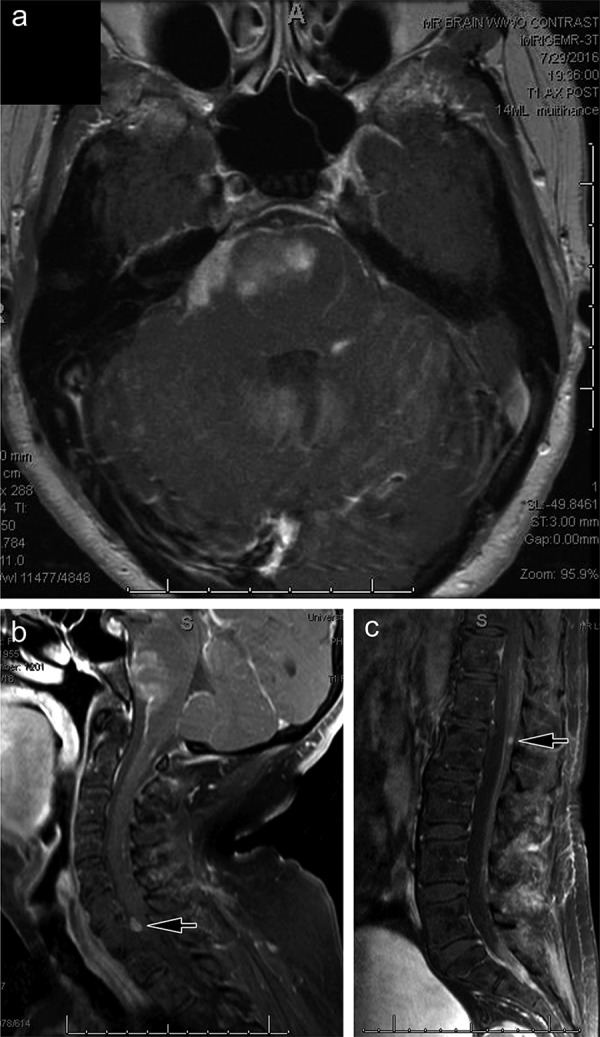
Adult diffuse midline gliomas can present with metastatic lesions. a: Contrast-enhanced axial T1-weighted MR image demonstrates a diffuse infiltrating lesion of the pons in a 61-year-old female patient (patient 10). b, c: Contrast-enhanced sagittal T1-weighted MR image of the spinal cord demonstrating leptomeningeal metastases at diagnosis (arrows).

Tumors involved thalamic (adult = 7; pediatric = 7), spinal cord (adult = 4; pediatric = 2), pons (adult = 1; pediatric = 6), and hypothalamic (n = 1) sites. Two of the adult spinal cord tumors originated in the conus medullaris (Table 1). In 2 patients, 1 adult and 1 pediatric, leptomeningeal dissemination had been noted at the time of clinical presentation and caused considerable diagnostic confusion. This was especially problematic in patient 10, a 61-year-old female with a pontine primary (Figure 1a) and spinal cord leptomeningeal metastases (Figure 1b, c). Two patients developed leptomeningeal dissemination within 1 year after diagnosis (Table 1). 

**Figure 2.**
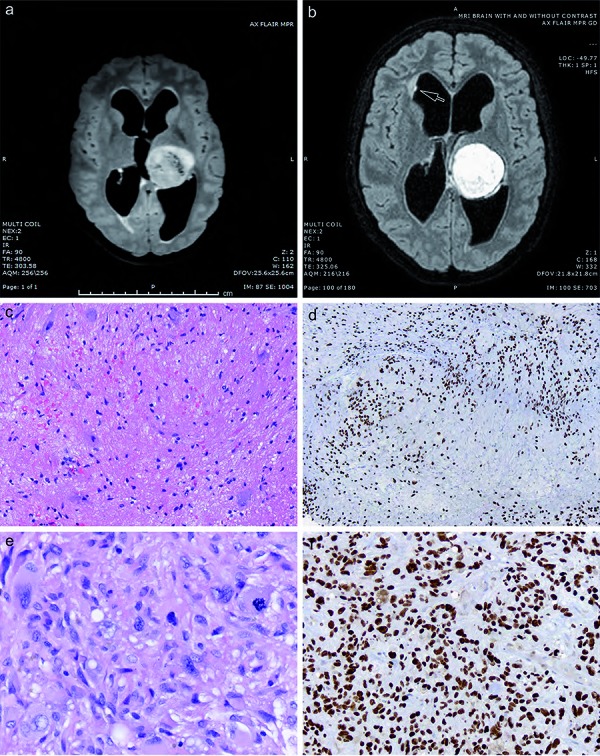
Aggressive nature of diffuse midline gliomas with *H3 K27M* mutation and ganglioglioma (GG) morphology, with early metastatic recurrence. a: Contrast-enhanced axial FLAIR image demonstrating a large left thalamic lesion (patient 28). b: Contrast-enhanced axial FLAIR image demonstrating primary thalamic lesion with a new finding of punctate focus of enhancement in the right lateral ventricle with adjacent white matter hyperintensity (arrow) demonstrating the development of metastatic disease within 3 months after diagnosis. c: This child originally showed a “pure” GG on biopsy, with irregularly sized and placed neuronal cells in a paucicellular background. d: However, the tumor was diffusely immunoreactive for nuclear H3 K27M, including the larger sized ganglionic tumor nuclei. e: Her recurrent tumor 3 months later no longer contained any areas of low grade GG; instead a highly pleomorphic glioblastoma (f) with diffuse H3 K27M immunostaining had developed. (c, e: H & E, × 200, × 400; d, f: *H3 K27M* immunostaining with light hematoxylin counterstain, × 200, × 400).

**Figure 3.**
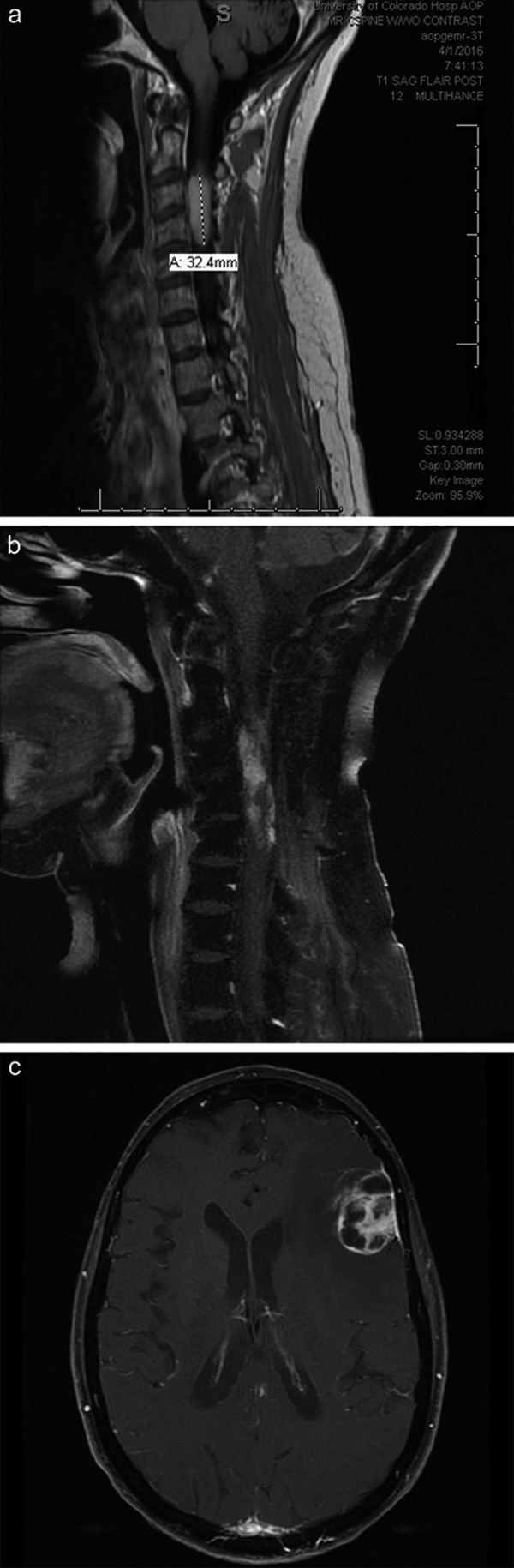
Aggressive nature of diffuse midline gliomas with *H3 K27M* mutation and pylotic astrocytome (PA) morphology, with later metastatic recurrence. a: Contrast-enhanced sagittal T1-weighted image demonstrating a cervical cord PA (patient 4) at presentation. b: Contrast-enhanced sagittal T1-weighted image demonstrating local progression within 1 year of diagnosis of the PA shown in (a). c: Contrast-enhanced axial T1-weighted image demonstrating additional large supratentorial leptomeningeal/dural metastasis with transformation of the PA to glioblastoma 1 year after diagnosis.

**Figure 4.**
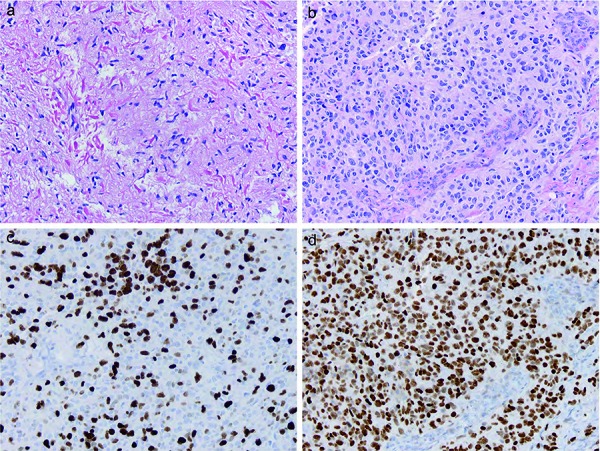
Aggressive nature of diffuse midline gliomas with *H3 K27M* mutation and pilotcytic astrocytoma (PA) morphology, with later metastatic recurrence. a: Patient 4 originally had a cervical cord pilocytic astrocytoma that was paucicellular and filled with innumerable eosinophilic elongated Rosenthal fibers, but was diffusely immunoreactive for H3 K27M. b: One year later the same patient’s supratentorial metastasis showed transformation to glioblastoma, with (b) hypercellularity and microvascular proliferation, (c) high MIB-1 labeling index, and (d) retention of diffuse H3 K27M immunoreactivity. (a, b: hematoxylin and eosin, × 200, × 200; c: MIB-1 immunostaining, × 200; d: H3 K27M immunostaining, × 200).

Four cases had other morphologies at initial biopsy, including pure GG (n = 3, pediatric) and PA (n = 1, adult) histologies. One of the GGs was a 16-year-old girl with an original biopsy demonstrating a pure thalamic GG (Figure 2a), but within a year she developed increased local recurrence (Figure 2b) with small ventricular metastases (Figure 2b). Her original tumor was a WHO grade I GG (Figure 2c) with diffuse H3 K27M immunostaining (Figure 2d), but on recurrence had transformed to glioblastoma with very pleomorphic cells (Figure 2e) and diffuse H3 K27M immunostaining (Figure 2f). The only adult patient with an alternate morphology was patient 4 with a cervical cord PA at presentation (Figure 3a) who progressed locally within a year (Figure 3b) and developed a large supratentorial leptomeningeal/dural metastasis (Figure 3c). Her original tumor was histologically a “pure” PA, WHO grade I, with innumerable characteristic Rosenthal fibers (Figure 4a) but diffuse H3 K27M immunostaining (Figure 4b). In contrast, her supratentorial metastasis showed glioblastoma transformation (Figure 4c) with diffuse H3 K27M immunostaining (Figure 4d). An additional pediatric patient, patient 23, had extensive calcifications in his tumor, but no GG features and several of the tumors were graded as WHO grade II (low-grade glioma) (Table 1). Six of the 13 assessed adult cases and 6 of the 15 pediatric cases showed nuclear p53 immunostaining in greater than 25% of tumor cells, acknowledging that firm cut-off levels for what percentage labeling index for nuclear p53 protein parallels *TP53* mutation have not been established. 

The known lower frequency of *ATRX* mutation/loss of ATRX nuclear immunostaining in only 10 – 15% of *H3 K27M-*mutant tumors [5] makes this feature less amenable to comparisons between the 2 cohorts. However, of the 7 adult cases tested for nuclear ATRX by IHC, nuclear ATRX was lost in 4 and retained in 3. For pediatric cases, of the 7 tested cases, it was lost in 1 and retained in 6. Limited size of the tested group also makes firm conclusions impossible. 

MIB-1 labeling indices correlated with the diagnosis and grade assigned prior to H3 K27M IHC testing (Table 1). For the adult cohort, 1 case met WHO criteria for diffuse astrocytoma, WHO grade II (MIB-1 < 1%), 7 cases met WHO criteria for anaplastic astrocytoma, WHO grade III (MIB-1 range 5 – 15%), and 1 was initially pilocytic astrocytoma, WHO grade I (MIB-1 very focally 6 – 7%); 5 were histologically glioblastoma, WHO grade IV (MIB-1 range 18 – 40%), based on the presence of mitotic activity plus either/both microvascular proliferation and necrosis. Amongst the pediatric cases, 1 met WHO criteria for diffuse astrocytoma, WHO grade I (MIB-1 3%), 3 met criteria for anaplastic astrocytoma, WHO grade III (MIB-1 range 6 – 10%), and 3 cases met WHO criteria for ganglioglioma, WHO grade I (MIB-1 < 1%); 8 were histologically glioblastoma, WHO grade IV (MIB-1 range 18 – 40%). The mean MIB-1 rate for glioblastomas in adults was 26% and for pediatric patients was 28%, i.e., was nearly identical. 

EGFR amplification had been assessed on 9 adult cases; 8 cases were negative and 1 case showed rare cells positive for EGFR amplification (case 9). Amongst adult cases, 7 were additionally assessed for loss of PTEN sequences relative to centromere 10; 5 cases were negative, 2 showed borderline loss (cases 7, 11), and 1 was positive (case 12). 

EGFR amplification had been assessed on 4 pediatric cases, all of which were negative and PTEN additionally assessed on 3, 1 of which (case 16) showed PTEN loss. Thus, EGFR amplification and PTEN loss were uncommon in either the adult or pediatric cohorts. 

Mean survival for adult patients was 9.3 months, with a median survival of 8.4 months (standard error 1.887). Mean survival for pediatric patients was 8.9 months, with a median survival of 6.86 months (standard error 1.537). Combined survival for the entire 28 patient cohort showed a mean survival of 9.1 months (standard error 1.180), with a median survival of 8.0 months. However, as noted in Table 1, occasional patients enjoyed slightly longer survival, including a young adult with a thalamic tumor (patient 3, 27.2 months) and 2 pediatric patients with thalamic tumors (patient 18, 19.8 months and patient 23, 18.2 months). In addition, patient 26 with a spinal cord GG showed longer survival (19.6 months), but patient 27, also with a GG, did not (9.6 months). The most recent case in our series was additionally a GG and although it has the shortest follow-up (patient 28, 2.8 months), local progression and ventricular metastases had already occurred (Figure 2). The adult with the PA has survived 16.2 months, but also has shown recent local progression and metastasis (Figures 3, Figure 4). Thus, the prognosis for these two may be guarded in the long term. 

## Discussion 

A strength of our study is that it comes from a single joint adult/pediatric institution and thus, unlike previously published case reports [6], provides perspective on how often other morphologies (3 GG, 1 PA, 2 LGG (6/28, 21%)) occur within an *H3 K27M*-mutant cohort. Our 28 cases are similar in number to the 21 *H3 K27M*-mutant adult patients recently published by Meyronet et al. [10], although that series was derived from multiple French institutions. Their study also identified “7 patients (33%) for whom pathological analysis hesitated between a diffuse glioma, ganglioglioma, or pilocytic astrocytoma” [10]. Other large series have also contained a significant percentage of cases with “alternate” morphology (n = 7/47 cases, 15%), including 2 with giant cells, and 1 each with pilomyxoid features, primitive neuroectodermal-like (PNET-like) foci, neuropil-like islands, ependymal areas + sarcomatous transformation, ganglionic + rhabdoid differentiation [3]. 

Indeed, if only looking at the individual case reports of mutant *H3 K27M*-mutant tumors with “pure” GG or PA features in the literature to date, an incorrect perception could be reached that such cases are quite exceptional, when in fact, they are not. This has important implications for institutions when assessing midline gliomas. Our data would support that all midline gliomas, in patients of all ages and with all glial morphologies, should be tested for H3 K27M by IHC. It is also becoming clear that even those with “pure” histological appearance of GG or PA do not demonstrate prolonged survival and should therefore be diagnosed as “diffuse midline glioma, *H3 K27M-*mutant” and given the associated grade of IV, even if occasional patients survive longer before undergoing recurrence, dissemination, and demise. 

Although we did not encounter *H3 K27M*-mutant tumors with ependymal-like areas or “pure” posterior fossa ependymomas with this mutation in our series, this is not surprising, as another recent series also did include such cases [10]. Out of 151 tested posterior fossa ependymomas, Ryall et al. [18] identified only a single case with this mutation. One could make the argument that this is so rare that testing of posterior fossa ependymomas is not cost-effective, but individual institutions will at this time have different practices based on the current literature. 

Our series is notable in that the adult cohort included several patients with very advanced age (72, 81 years). While an 82-year-old female was reported by Meyronet et al. [10], the mean age in their adult series was 2 decades younger than ours (32 years vs. our 52 years). Their median survival in adults (19.6 months) was considerably longer than ours (8.4 months), underscoring the need for more data on adult cohorts. 

Our adult cohort was also notable for the inclusion of one of the two very recently-reported examples of H3 K27M mosaicism [21]. These cases showed mosaicism by IHC and had been extensively studied genetically. When this unusual IHC pattern first was recognized at the time of diagnosis, the mosaicism prompted some discussion as to whether such a case warranted the WHO diagnosis of diffuse midline glioma, *H3 K27M-*mutant, WHO grade IV at both of the two different institutions at which the patient was seen. This patient’s short survival of 6.0 months would suggest that this mosaicism can carry the same adverse prognosis as that displayed by ordinary, diffuse midline gliomas, *H3 K27-*mutant, WHO grade IV with diffuse immunostaining. 

Unlike almost all previously reported series, we were interested in comparing all aspects of pediatric vs. adult patients with H3 K27M tumors. Our cohort included both 1 adult and 1 pediatric patient with pontine *H3 K27M-*mutant diffuse gliomas with leptomeningeal dissemination at first presentation, a behavior well-recognized to occur late in the clinical course of DIPGs [4, 22, 23], but less commonly at first admission. We further document that 1 adult PA and 1 pediatric GG had clinical and histological progression to high-grade tumors within a year of diagnosis. One spinal cord GG did show more prolonged survival of 19.6 months, however. 

Longer survival has occasionally been demonstrated in some *H3 K27M-*mutant tumors with other histological morphologies. A 7-year-old girl with an *H3 K27M-*mutant PA, WHO grade I, survived 10 years before undergoing malignant transformation, with demise within 1.5 years after progression [6]. A 12-year-old girl with a *K27M-*mutated thalamic GG, WHO grade I, was stable for 7 years before relapse showed an anaplastic GG, WHO grade III; she developed local progression and leptomeningeal dissemination despite chemotherapy and died 14 months after second surgery [7]. A second patient in the same study, a 25-year-old male with a cerebellar GG with PA elements showed rapid progression histologically and clinically within 2 months, with demise 33 months after initial presentation [7]. Of the 5 pediatric midline GGs in the study by Pages et al. [12] (1 previously reported, above), 1 was alive at 7 years, and 2 more at 9 months and 1 year post diagnosis. 

Although a very recent paper detailing outcome in 65 pediatric tumors with mutation found a “uniformly adverse prognosis” [24], this study specifically included only tumors occurring in patients ages 0 – 18 years and “showing features either of a diffuse midline glioma, *H3 K27M-*mutant, WHO grade IV (DMGIV), a midline glioblastoma WHO grade IV (GBMIV), or a midline anaplastic astrocytoma WHO grade III” [24]. Other histological morphologies were not included. 

Our study further adds to the literature important information especially about adults with mutant tumors. In our study, adult and pediatric patients with *H3 K27M*-mutant tumors showed nearly identical adverse prognosis, with mean survival of 9.3 and 8.9 months, respectively. The 4 patients with other “pure” GG or PA morphologies did not fare as well as several in the literature. Some investigators have suggested that for *H3 K27M*-mutant tumors with GG morphology that “cases should not be graded and treated as grade IV tumors because they have a better spontaneous outcome” [12]. However, accumulating data since that time from newer cases, including the 4 in our current study, indicates that prognosis for individual patients with midline GG or PA histology plus *H3 K27M* mutation is highly variable and currently unpredictable. Thus, further documentation of the expanding range of clinical, histological, and survival features in *H3 K27M*-mutant midline, and even rarely, non-midline [25] or mosaic [21] tumors is important. 

Limitations of our study do exist. We, like most laboratories, utilized IHC for identification of *H3 K27M* mutations, rather than direct genetic testing. The commercially available antibody for IHC detection of H3.3K27M has been shown to be highly effective at identifying these tumors with a reported 100% sensitivity, specificity, and positive predictive value (PPV) [20]. IHC has also been successfully utilized across a spectrum of tumors with variations in morphology and did not produce false negatives in tumors in the cerebral hemispheres [3]. These data would support the expanded use IHC for the clinical identification of H3 K27M mutations clinically. The limitation arises in that the antibody is positive not only with *H3 K27M* mutation but also with the *H3.1 K27M* mutation; it is not positive in patients with an *H3.3G34R* mutation. All of these mutations are anticipated to lead to global reductions in H3K27me3. 

## Acknowledgment 

The authors thank Mrs. Diane Hutchinson for manuscript preparation and Ms. Lisa Litzenberger for photographic expertise. 

## Funding 

Support provided by: NIH/NCI (K08CA193982), and the Morgan Adams Foundation (JML). 

## Conflict of interest 

The authors have no conflicts to declare 

**Table 1.** Demographics, histological, and survival data for adult vs. pediatric patients.

Patient Age (y)/gender	Anatomical location	p53/MIB-1 immunostaining	Diagnosis by histological criteria alone, prior to H3 K27M IHC	Died of disease (DOD) vs. living at time of publication	Survival (months)
Adult patients					
1) 33 years, female	Conus medullaris	> 40% 15%	Anaplastic astrocytoma, WHO grade III	DOD	5.1
2) 27 years, female	Thoracic T8-12	Negligible 20%	Glioblastoma, WHO grade IV	DOD	10.2
3) 31 years, male	R. thalamus	> 50% 8%	Anaplastic astrocytoma, WHO grade III	DOD	27.2
4) 49 years, female	Cervical C4	Not done focally 6 – 7%	Pilocytic astrocytoma, WHO grade I (pure)	Living	16.2
4) relapse	Cerebral meninges/dura	Negligible 35%	Glioblastoma, WHO grade IV	Living	16.2
5) 69 years, male	L. thalamus	< 8% 5%	Anaplastic astrocytoma, WHO grade III H3 K27M mosaicism	DOD	6
6) 28 years, female	R. thalamus	Negligible 1%	Diffuse astrocytoma, WHO grade II	Living	12.7
7) 52 years, male	L. thalamus	> 90% 12%	Anaplastic astrocytoma, WHO grade III	Living	10.8
8) 33 years, male	R. thalamus	Negligible 6.5%	Anaplastic astrocytoma, WHO grade III	Living	11
9) 72 years, female	Conus medullaris	> 50% 18%	Glioblastoma, WHO grade IV	DOD	3.2
10) 61 years, female	Pons, leptomeningeal spread at presentation	< 8% 25%	Glioblastoma, WHO grade IV	DOD	9.4
11) 81 years, female	Hypothalamus	< 5% 40%	Glioblastoma, WHO grade IV	DOD	1.9
12) 72 years, female	R. thalamus	> 40% 6.5%	Anaplastic astrocytoma, WHO grade III	Living	8.4
13) 64 years, female	R. thalamus	> 30% 15%	Anaplastic astrocytoma, WHO grade III	Living	2.3
Pediatric patients					
14) 12 years, female	R. thalamus	> 60% 18%	Glioblastoma, WHO grade IV	Living	5.5
15) 3 years, female	Pons	15 – 20% 30%	Glioblastoma, WHO grade IV	Living	5.0
16) 14 years, male	Pons	> 90% 25%	Glioblastoma, WHO grade IV	DOD	2.9
17) 6 years, female	Pons, leptomeningeal spread at presentation	Negative 20%	Anaplastic astrocytoma, WHO grade III	DOD	6.9
18) 11 years, male	L. thalamus	> 40% 35%	Glioblastoma, WHO grade IV	Living	19.8
19) 6 years, male	R. thalamus	> 95% 3%	Diffuse astrocytoma, WHO grade II	Living	10.1
20) 7 years, male	Pons	11% 6%	Anaplastic astrocytoma, WHO grade III	DOD	3.9
21) 3 years, female	R. thalamus	~ 100% 10%	Anaplastic astrocytoma, WHO grade III	DOD	3.2
22) 12 years, female	Pons	> 60% 35%	Glioblastoma, WHO grade IV	DOD	5.9
23) 3 years, male	R. thalamus	Negligible 8%	Anaplastic astrocytoma, WHO grade III, with extensive calcifications	Living	18.2
24) 8 years, female	Cervico-medullary junction	< 5% 40%	Glioblastoma, WHO grade IV	DOD	9.7
25) 12 years, male	Pons	Negligible ND	Glioblastoma, WHO grade IV	DOD	11.1
26) 6 years, female	Spinal cord	7 – 8% 1%	Ganglioglioma, WHO grade I	DOD	19.6
27) 13 years, male	Pons	Negligible 1%	Ganglioglioma, WHO grade I	DOD	9.6
28) 16 years, female	L. thalamus	Not done 1%	Ganglioglioma, WHO grade I	Living	2.8
28) relapse	L. thalamus leptomeningeal spread at recurrence	> 90% 1%	Glioblastoma, WHO grade IV	Living	2.8
